# Correlation between Neutrophil-to-Lymphocyte Ratio, Platelets-to-Lymphocyte Ratio, C-Reactive Protein-to-Albumin Ratio and Clinical Picture of Elderly Chronic Heart Failure Patients

**DOI:** 10.3390/jcm13020433

**Published:** 2024-01-12

**Authors:** Tomasz Powrózek, Aneta Skwarek-Dziekanowska, Grzegorz Sobieszek, Teresa Małecka-Massalska

**Affiliations:** 1 Department of Human Physiology of the Chair of Preclinical Sciences, Medical University in Lublin, 20-080 Lublin, Poland; tmalecka@gmail.com; 2 Department of Cardiology, 1st Military Clinical Hospital with the Outpatient Clinic, 20-049 Lublin, Poland; anetask@gmail.com (A.S.-D.); grzes.bies@interia.pl (G.S.)

**Keywords:** chronic heart failure, CAR, NLR, PLR

## Abstract

Background: Neutrophil-to-lymphocyte ratio (NLR), platelets-to-lymphocyte ratio (PLR) and C-reactive protein-to-albumin ratio (CAR) are believed to be potential inflammatory markers that are closely related to the prognosis and course of cardiovascular diseases. The main goal of this study was the evaluation of NLR, PLR and CAR as factors reflecting the clinical picture and the prognosis of elderly chronic heart failure (CHF) patients. Methods: In 150 elderly patients with newly diagnosed CHF, the NLR, PLR and CAR were correlated with cardiac, laboratory and nutritional parameters. Results: Systemic inflammatory ratios were correlated with selected patient’s parameters. CAR was associated with an unfavorable clinical picture of CHF—a reduced EF (*p* = 0.007), an elevated PASP (*p* = 0.014), an increased LVESD in both males and females (*p* = 0.032 and 0.024, respectively) and a decreased TAPSE (*p* = 0.023). CAR allowed us to distinguish between NYHA I–III and NYHA IV classes with AUC of 0.830. By analyzing the five-year mortality rate in patients with different CAR values, the greater death rate was recorded for patients with high CAR values—one-year death rate (40.3% vs. 17.2%) and five-year death rate (80% vs. 58.3%) (*p* = 0.002). Both NLR and PLR correlated only with selected parameters. Conclusion: An analysis of inflammatory markers, mainly CAR, allows the management of CHF, because its value can reflect the cardiac and nutritional status of patients with a prognostic value. NLR and PLR can serve as supplementary examinations for CAR evaluation.

## 1. Introduction

Chronic heart failure (CHF) is a complex disease that involves cardiac dysfunction frequently followed by systemic disorders and multiple organ failure [1,2]. CHF is one of the leading causes of death globally, mainly in elderly patients (aged over 65 years), and it is characterized by a negative impact on a patient’s quality of life and the reduced level of physical activity in daily life. According to the results of available meta-analyses, the estimated five-year survival rate in geriatric CHF individuals is less than 50% [3,4].

Systemic inflammation is considered as a key factor influencing CHF development. However, the connection between CHF and inflammatory response is complex and involves a relationship network between heart and different internal organs (the multiorgan hypothesis of CHF) [5]. The mechanism of inflammatory activation can be caused by hemodynamic stress (e.g., overload of the left heart ventricle), the coexistence of other systemic disorders (diabetes, obesity, and atherosclerosis), humoral activity, and imbalances in muscles and the gastrointestinal system [5,6]. As a result of inflammatory response due to the pro-inflammatory cytokines releases, such as tumor necrosis factor alpha (TNF-α) or interleukins (IL-1 and IL-6), released from different tissues, the systolic and diastolic dysfunction of cardiomyoctes and their atrophy and remodeling is caused [7,8].

Recent studies that have widely analyzed patients suffering from different systemic diseases have inferred that neutrophil-to-lymphocyte ratio (NLR), platelets-to-lymphocyte ratio (PLR) and C-reactive protein-to-albumin ratio (CAR) are potential inflammatory markers that are closely related to the prognosis of cardiovascular diseases [9,10]. They have been also proposed the cost-effective, easily obtainable and available markers of systemic inflammation [11,12]. The above-mentioned benefits of NLR, PLR and CAR encourage their evaluation in daily clinical practice. To the best of our knowledge, there is a lack of significant literature data on NLR, PLR and CAR in elderly CHF patients and even in other CHF patients. The main goal of the study was the evaluation of NLR, PLR and CAR as factors reflecting the clinical picture and the prognosis of elderly CHF patients.

## 2. Materials and Methods

A total of 150 elderly patients (85 men and 65 women; median age: 72 years; range: 65–94 years) with newly diagnosed CHF were retrospectively analyzed. All study participants were recruited for the research at the Clinic of Cardiology and Internal Medicine, 1st Military Hospital in Lublin, Poland, between 2013 and 2015. Criteria issued by the European Society of Cardiology were applied in order to diagnose CHF. It included an analysis of patients’ medical history, physical examination, laboratory testing and echocardiographic examination. The extent of the disease was assessed by the New York Heart Association (NYHA) functional classification (class I–IV, according to the disease severity). The parameters derived from echocardiography (with the use of Vivid™ T8 Ultra Edition, GE Healthcare, Chicago, IL, USA) that were analyzed are as follows: EF%—ejection fraction of the left ventricle; LVEDD and LVESD—left ventricular end-diastolic and end-systolic diameters; PASP—pulmonary artery systolic pressure; and TAPSE—tricuspid annular plane systolic excursion. Laboratory testing included the measurement of a number of circulating neutrophils, lymphocytes, and platelets as well as the concentration of serum albumin, NT-proBNP, hemoglobin and inflammatory markers: CRP, IL-6 and TNF-α. CAR was calculated by dividing the serum CRP concentration (mg/dL) by the serum albumin concentration (g/dL), NLR by dividing the absolute count for neutrophils (×10^9^/L) by the absolute count for lymphocytes (×10^9^/L), and PLR by dividing the absolute count for platelets (×10^9^/L) by the absolute count for lymphocytes (×10^9^/L). All laboratory parameters were analyzed during the routine examination of patients with the use of Roche Cobas 6000 (Basel, Switzerland), and Cormay Mythic 70 (Lublin, Poland). Inclusion and exclusion criteria were defined for study participants. Inclusion criteria were as follows: newly diagnosed CHF, age > 65 years, provided signed informed consent, and Polish ethnicity. Exclusion criteria were as follows: the presence of acute coronary syndrome, thyroid disorders (neither hypo- nor hyperthyroidism), implanted cardioverter defibrillator and coronary artery bypass grafting. The nutritional examination of the patients was conducted with the use of clinical questionnaires (Subjective Global Assessment (SGA) and Nutritional Risk Score (NRS-2002)) and the analysis of the body composition using bioimpedance analysis (BIA) that included the measurement of fat mass (FM) and fat-free mass (FFM). The ImpediMed bioimpedance analysis SFB7 BioImp v1.55 device (Pinkenba, QLD, Australia) was used to measure parameters reflecting the body composition. The study protocol was approved by the local ethics committee in Medical University in Lublin, Poland (no. of consent: KE-0254/64/2017). The baseline characteristic of studied CHF patients are presented in Table 1.

### Statistical Analysis

MedCalc computer software (MedCalc, Ostend, Belgium), version 18.5, was used for statistical purposes. Differences in CAR, NLR and PLR values were compared between groups of patients according to the clinical, echocardiographic, laboratory and nutritional parameters using Mann–Whitney U test. The cut-off values of parameters were determined based on the literature data, clinical criteria or their median values. CAR, NLR and PLR values were presented as median scores with an interquartile range (IQR). Investigated ratios were correlated with different patient parameters with the use of Spearman’s rank correlation (Spearman’s rho). Receiver operating curve (ROC) analysis with the calculation of area under the ROC (AUC) was applied to assess CAR, NLR and PLR diagnostic values in order to distinguish between patients qualified to different NYHA classes. In order to select the predictors of an NYHA class, multiple regression analysis was carried out. Kaplan–Meier estimator (univariate) was used to analyze the independent prognostic values of CAR, NLR and PLR for patients’ overall survival (OS). Cox proportional-hazard analysis (multivariate) was applied to select factors affecting OS in the studied CHF patients. Statistically significant results of the used tests demonstrated *p*-values below 0.05 (*p* < 0.05).

## 3. Results

The median values of NLR, PLR and CAR in the studied group were as follows: 3.08 (2.19–4.44), 126.0 (90.1–162.1) and 1.76 (0.57–7.42), respectively. In Table 2, the median values of NLR, PLR and CAR were compared between patients with different clinical–demographic and echocardiographic parameters. The most significant results were recorded for CAR. Its value was higher in the following group of patients with NYHA III–IV (median: 4.74 vs. 1.03; *p* < 0.001), a reduced EF (median: 2.65 vs. 1.16; *p* = 0.007), an elevated PASP (2.96 vs. 1.13; *p* = 0.014), an increased LVESD in both males and females (*p* = 0.032 and 0.024, respectively) and a decreased TAPSE (median: 3.41 vs. 1.35; *p* = 0.023). Moreover, higher CAR values were associated with the presence of both dyspnea at rest (median: 3.61 vs. 1.13; *p* < 0.001) and cardiac arrhythmias (median: 2.87 vs. 1.13; *p* = 0.016). Regarding the higher NLR values, similar results were noticed for NYHA grade (median: 3.71 vs. 2.65; *p* = 0.002), PASP (median: 3.19 vs. 2.63; *p* = 0.016), TAPSE (median: 3.42 vs. 2.89; *p* = 0.040) and the occurrence of dyspnea at rest (median: 3.75 vs. 2.66; *p* < 0.001). As for elevated PLR, the significant results were achieved for age > 72 years (median: 138.9 vs. 107.9; *p* = 0.036), NYHA grade (median: 139.0 vs. 110.6; *p* = 0.042) and the presence of dyspnea at rest (median: 141.0 vs. 112.5; *p* = 0.028).

Higher values of NLR and CAR were found in patients with the presence of elevated concentrations of serum inflammatory markers—CRP, IL-6, TNF-α, and NT-proBNP (all *p* < 0.05). Unlike both CAR and NLR, PLR was associated only with a higher serum CRP concentration (*p* = 0.046). Noticeably, both PLR (median: 146.6 vs. 110.6; *p* = 0.007) and CAR (median: 4.79 vs. 1.24; *p* = 0.002) were increased in patients with anemia. Investigating the relationship between studied markers and parameters reflecting nutritional status of the patients, we found their interdependencies. CAR and NLR were significantly higher in patients with hypoalbuminemia (*p* < 0.001 and *p* = 0.004, respectively) and individuals with a higher NRS-2002 score (both *p* < 0.001). Only CAR corresponded with SGA, and significantly higher CAR values were observed in PNS patients qualified as mildly (B) or severely malnourished (C) in contrast to those of well-nourished ones (A) (median: 3.57 vs. 0.96; *p* < 0.001). Regarding the body composition parameters, NLR was elevated in patients with lower FM (median: 3.59 vs. 2.67; *p* = 0.016), and PLR was elevated in patients with lower FFM (145.3 vs. 104.3; *p* = 0.011). Differences in NLR, PLR and CAR values depending on nutritional and laboratory parameters are presented in Table 3.

**Figure 1:**
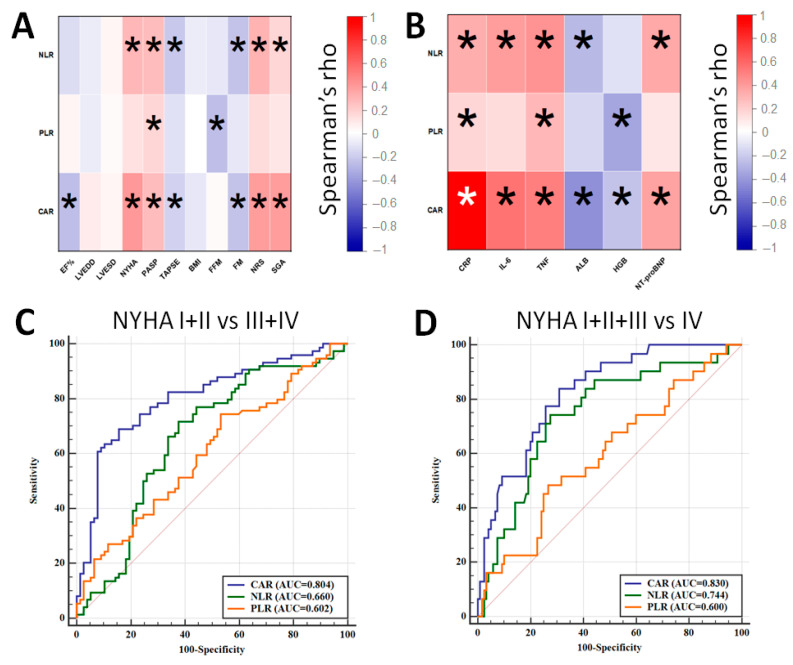
Correlation between NLR, PLR, CAR and echocardiographic and nutritional parameters (**A**) and laboratory results (**B**). ROC curves demonstrating the diagnostic accuracy of NLR, PLR and CAR for distinguishing between NYHA I + II and NYHA III + IV patients (**C**) and between NYHA I–III and NYHA IV patients (**D**) (*—statistically significant results).

Then, NLR, PLR and CAR were correlated with the studied parameters. Figure 1A demonstrates the result of the correlation between NLR, PLR, CAR and echocardiographic and nutritional factors. The strongest positive correlation for CAR was observed with NYHA (rho = 0.413), and its strongest negative correlation was observed with EF% (rho = −0.263), whereas for NLR, its strongest positive correlation was observed with NRS (rho = 0.301), and its strongest negative correlation was observed with FM (rho = −0.224), respectively. PLR significantly correlated only between PASP (rho = 0.178) and FFM (rho = −0.246). Regarding the laboratory examination, despite the obvious correlation between CAR and both CRP and albumin, this parameter most positively correlated with IL-6 (rho = 0.556) and most negatively correlated with hemoglobin concentration (rho = −0.230). The strongest positive correlation for NLR was found with TNF-α (rho = 0.426), and its strongest negative correlation was found with albumin concentration (rho = −0.265). Noticeable positive and negative correlations for NLR were found with PLR and TNF-α (rho = 0.300) and hemoglobin concentration (rho = −0.345), respectively. Figure 1B demonstrates the result of correlation between the values of NLR, PLR, CAR and studied laboratory parameters.

The next goal of the analysis was to assess the diagnostic accuracy of the studied parameters for distinguishing between patients qualified to NYHA I + II and NYHA III + IV classes as well as NYHA I–III and NYHA IV classes (Figure 1C,D). The best diagnostic accuracy was achieved for CAR as it allowed to distinguish between NYHA I + II and NYHA III + IV with 69.9% sensitivity and 84.6% specificity (AUC = 0.804; cut-off: 2.65) as well as between NYHA I–III and NYHA IV with a sensitivity of 83.9% and a specificity of 70.0% (AUC = 0.830; cut-off: 2.65). A combination of CAR with either NLR or NLR + PLR did not improve the diagnostic accuracy of the test.

Multiple regression model analysis selected the predictors of the higher NYHA classification in the studied group. Among the studied parameters, a low EF% (*p* < 0.001), the presence of dyspnea at rest (*p* < 0.001), a high CAR score (*p* = 0.029) and a high concentration of NT-proBNP (*p* = 0.031) were considered as significant predictors of higher NYHA classification—Table 4.

**Figure 2:**
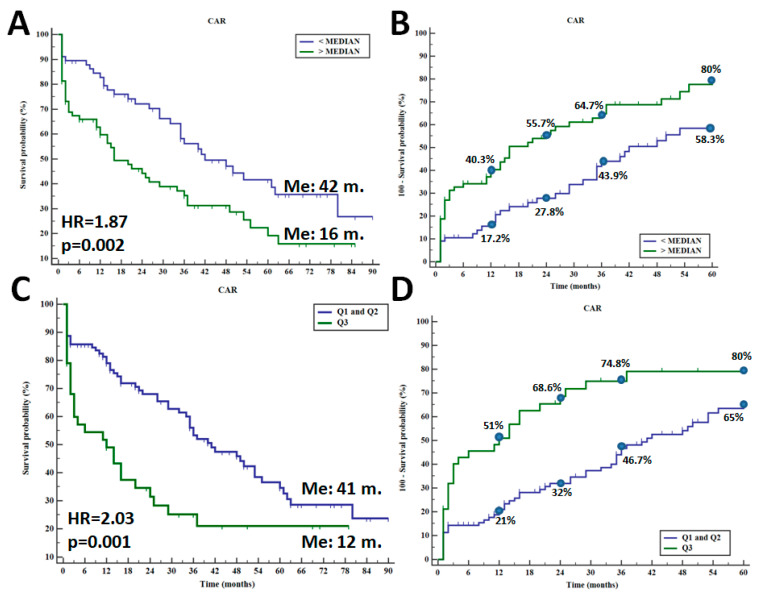
Overall survival in CHF patients depending on median CAR value (**A**) and five-year death rate (**B**) as well as overall survival in CHF individuals with CAR value between Q1 + Q2 and Q3 (**C**) and five-year death rate (**D**).

Among the studied parameters, only CAR significantly affected patients’ survival. Applying median CAR value as the criterion distinguishing between its high and low value, we observed a significantly shorter overall survival (OS) time in individuals with a CAR value above the median score (median OS: 42 months vs. 16 months; HR = 1.87; *p* = 0.002—Figure 2A). By analyzing the five-year mortality rate in patients with different CAR values, the greater death rate was recorded for patients with high CAR values—one-year death rate (40.3% vs. 17.2%) and five-year death rate (80% vs. 58.3%) (*p* = 0.002)—Figure 2B. The more substantial differences in OS were observed between patients with CAR value > Q3 (CAR > 7.4) compared to Q1 or Q2 values (median OS: 41 months vs. 12 months; HR = 2.03; *p* = 0.001—Figure 2C). In this model, one-year death rate was 51% vs. 21%, and five-year death rate was 80% vs. 65%, respectively (*p* = 0.001). A combination of CAR with either NLR or PLR did not improve the prognostic value of CAR. The impact of NLR and PLR as well as their combination with CAR are demonstrated in Supplementary Figures S1 and S2.

All the studied factors including CAR, NLR, PLR, echocardiographic, clinical, laboratory and nutritional parameters were introduced into the Cox proportional-hazard model. As a result, the serum albumin concentration (HR = 1.61; *p* = 0.032), the CAR value (HR = 1.23; *p* = 0.040), patient’s age (HR = 1.10; *p* = 0.042) and EF% (HR = 1.10; *p* = 0.044) were selected as significant dependent factors affecting the survival in the studied group of CHF patients.

## 4. Discussion

For clinical purposes, cost-effective, easily obtainable and available markers of systemic inflammation are desirable in order to assess the level of inflammatory response and predict the course of the disease. To date, NLR, PLR and CAR were studied in different populations of patients, including in patients with chronic diseases, inflammatory-related diseases and cancer [13,14,15,16,17]. Most of the studies investigating these markers in cardiovascular diseases have focused on heart failure (HF) patients. However, its exact clinical utility for CHF patients is still unknown. It is also worth underlining that most of the available studies regarding cardiovascular diseases have focused on the prognostic value of the inflammatory markers. We have correlated their values with various factors reflecting patients’ clinical picture, such as echocardiographic and nutritional parameters.

Recently, Öztürk et al. found a higher NLR value in patients with a reduced LVEF compared to that of patients with preserved or mildly reduced LVEF (*p* = 0.002); however, their study demonstrated a poor diagnostic accuracy to distinguish between patients with different LVEF phenotypes (AUC < 0.60) [18]. Also, Durmus et al. investigated NLR and PLR in HF patients and observed the inverse correlation between NLR and LVEF (rho = −0.409), but not for PLR. ROC analysis demonstrated adequate accuracy for predicting the presence of HF with an AUC of 0.868, but PLR recorded an AUC of 0.689 [19]. In another study, the NLR and PLR were also found to be inversely correlated with LVEF, but only PLR served as a predictor for HF (AUC = 0.76) [20]. In our study, only CAR inversely correlated with LVEF (rho = −0.263), and its value was significantly higher in patients with reduced LVEF when compared to that of individuals with mildly or preserved LVEF (median CAR: 2.65 vs. 1.16; *p* = 0.007). Tamaki et al. found in patients suffering from acute decompensate HF, a relationship between both high NLR and PLR values and parameters reflecting a cardiac condition. Patients demonstrating high values of NLR and PLR had an increased PASP (*p* < 0.001), an increased NT-proBNP concentration (*p* < 0.001) and a decreased hemoglobin concentration (*p* < 0.001) in contrast to those of patients with other NLR and PLR values [21]. Our results are consistent with these findings, and we found higher values of CAR and NLR in patients with elevated PASP and increased NT-proBNP. Higher values of NLR and PLR were noticed in patients with anemia. Interestingly, we recorded significantly higher values of CAR, NLR and PLR in patients qualified to NYHA III-IV class (all *p* < 0.05). We have not found a relationship between the studied inflammatory markers and NYHA in the literature data so far. The best diagnostic accuracy to distinguish between patients with various NYHA classes was demonstrated by CAR—an AUC of 0.804 for NYHA I + II vs. III + IV and an AUC of 0.830 for NYHA I–III vs. IV. We also selected a lower EF% (*p* < 0.001), the presence of dyspnea at rest (*p* < 0.001), a high CAR score (*p* = 0.029) and the high concentration of NT-proBNP (*p* = 0.031) as significant predictors of higher NYHA classification. The higher CAR values were also noticed in patients with worse nutritional statuses (B and C according to the SGA and ≥3 according to the NRS-2002). Considering these results, it can be inferred that CAR can be a valuable marker for the selection of patients with poor clinical picture and severe symptoms of CHF. However, its clinical significance in HF and CHF is limited only to the investigation of its prognostic value.

A large data set derived from the study of Yang et al. indicated the role of high CAR as a risk factor of cardiovascular disease development, even better than CRP or albumin [22]. As for the prognostic value of CAR, recently, Sonsöz et al. investigated CAR in acute HF and noticed an increased mortality in the follow-up of the group of patients with high CAR values when compared with those of other patients (death rate: 36.7% vs. 12%; *p* < 0.001), and the HR was 1.69; *p* = 0.042 [23]. In another study that enrolled outpatients with HF, a high CAR value was selected as an unfavorable prognostic factor both by univariate and multivariate survival analysis (HR = 2.80 and HR = 2.13, respectively). In the group of survivors, 38% of HF patients had a CRP value over the median score, whereas in the non-survivor group, it was 95% (*p* < 0.001) [24]. In our research, we found the prognostic value only for CAR, but not for NLR or PLR. CHF patients with CAR values over the median score had worse prognosis than others (univariate median OS: 42 months vs. 16 months; HR = 1.87; *p* = 0.002; and multivariate HR = 1.23; *p* = 0.040). By analyzing the manner of CAR stratification, the five-year death rate was found to be 80% and 58.3%, respectively. Changing the manner stratification, patients with CAR value over the Q3 had five-year death rate of 80% vs. 65%, respectively (HR = 2.03). However, we did not achieve significant results for NLR or PLR, and the literature reports are not consistent; however, in most of them, both high NLR and PLR negatively affected HF patients’ survival. Lu et al. investigated the role of PLR in over 1200 HF patients and did not find a relationship between its value and either the mortality risk or the survival time of the patients. Regarding NLR, it demonstrated a prognostic value only when its value was at the highest quartile (HR = 1.59). Additionally, it demonstrated a rather low diagnostic accuracy for predicting death due to HF, with AUCs of 0.58 and 0.64, respectively [25]. A recent comprehensive review showed that PLR is not consistently an independent predictor for survival in HF, and this is probably related to the different cut-off values proposed by different authors [26].

## 5. Conclusions

The studied inflammatory markers, i.e., CAR, NLR and PLR, are easy to evaluate in clinical conditions, and they demonstrate utility for the evaluation of elderly CHF patients’ clinical condition. They correlate with the level of inflammatory markers, the NYHA class, and the selected cardiac and nutritional parameters. Among them, CAR seems to be most valuable due to its correlation with a large number of the studied parameters, its diagnostic accuracy for the NYHA class and its prognostic value. The obtained results underline the utility of inflammatory markers’ assessment in CHF patients. Our results are not free from limitations. We have evaluated selected inflammatory markers in a relatively small group of patients in a retrospective manner; thus, further studies should deal with these limitations.

## Figures and Tables

**Table 1:** Baseline characteristics of CHF patients.

Factor		*n* = 150; 100%
**Gender**	Male	85 (56.7%)
	Female	65 (43.3%)
**Age**	Median (range)	72 years (65–94 years)
	<72 years	73 (48.7%)
	≥72 years	77 (51.3%)
**Diabetes mellitus**		25 (16.7%)
**Hypertension**		47 (31.3%)
**Hyperlipidemia**		56 (37.3%)
**Dyspnea at rest**		53 (35.3%)
**Exertional dyspnea**		136 (90.7%)
**Cardiac arrhythmias**		70 (46.7%)
**NYHA**	I–II	74 (49.3%)
	III–IV	76 (50.7%)
**Echocardiographic parameters**		
**EF%**	Median (IQR)	42 (29–55)
**PASP (mmHg)**	Median (IQR)	40 (32–48)
**LVESD (mm)**	Median (IQR)	42 (37–50)
**LVEDD (mm)**	Median (IQR)	54 (46–62)
**TAPSE (mm)**	Median (IQR)	19 (15–20)
**Laboratory testing**		
**ALB (mg/dL)**	Median (IQR)	3.50 (3.0–3.80)
**HGB (g/dL)**	Median (IQR)	13.2 (11.5–14.5)
**NT-proBNP (pg/mL)**	Median (IQR)	2827 (1237–5444)
**CRP (mg/dL)**	Median (IQR)	5.95 (2.0–21.55)
**IL-6 (pg/mL)**	Median (IQR)	7.0 (1.98–15.53)
**TNF-α (pg/mL)**	Median (IQR)	4.24 (3.13–5.76)
**Nutritional parameters**		
**BMI (kg/m^2^)**	Median (IQR)	28.0 (25.30–32.33)
**FM (kg)**	Median (IQR)	25.80 (18.34–32.70)
**FFM (kg)**	Median (IQR)	53.74 (47.55–62.30)
**SGA**	A	69 (46%)
	B–C	81 (54%)
**NRS-2002**	<3	108 (72%)
	≥3	42 (28%)

ALB—albumin; EF%—ejection fraction; FFM—fat-free mass; FM—fat mass; HGB—hemoglobin; LVEDD—left ventricular end-diastolic diameter; LVESD—left ventricular end-systolic diameter; NYHA—New York Heart Association; NRS-2002—Nutritional Risk Score; PASP—pulmonary artery systolic pressure; SGA—Subjective Global Assessment; and TAPSE—tricuspid annular plane systolic excursion.

**Table 2:** Differences in NLR, PLR and CAR values depending on patients’ clinical–demographic data and echocardiographic parameters.

Factor	NLR (IQR)	*p*	PLR (IQR)	*p*	CAR (IQR)	*p*
**Male**	3.34 (2.27–5.24)	0.283	123.0 (92.6–159.9)	0.749	2.06 (0.80–8.23)	0.218
**Female**	2.99 (1.97–4.78)		130.1 (87.2–167.8)		1.58 (0.47–7.39)	
**Age < 72 years**	2.71 (1.97–4.16)	0.109	107.9 (89.3–150.4)	***0.036***	1.23 (0.49–6.64)	0.194
**Age > 72 years**	3.19 (2.40–4.77)		138.9 (95.0–173.2)		2.15 (0.71–7.45)	
**NYHA I–II**	2.65 (1.87–3.79)	***0.002***	110.6 (86.54–152.5)	***0.042***	1.03 (0.40–2.49)	<***0.001***
**NYHA III–IV**	3.71 (2.60–5.09)		139.0 (97.6–167.9)		4.74 (1.11–9.93)	
**EF% < 40**	3.25 (2.48–5.04)	0.134	115.3 (92.6–161.9)	0.832	2.65 (0.79–9.65)	***0.007***
**EF% > 40**	2.80 (1.98–4.10)		131.1 (89.2–165.8)		1.16 (0.48–3.58)	
**PASP > 36 mmHg**	3.19 (1.81–4.16)	***0.016***	124.8 (96.2–165.9)	0.393	2.96 (0.82–9.39)	***0.014***
**PASP < 36 mmHg**	2.63 (2.61–4.99)		129.9 (80.7–157.5)		1.13 (0.47–3.55)	
**LVEDD** **(male < 56 mm)**	3.44 (2.59–6.26)	0.396	145.9 (93.5–178.1)	0.119	2.18 (0.83–6.28)	0.903
**LVEDD** **(male > 56 mm)**	3.21 (2.20–5.08)		111.8 (90.1–157.1)		1.73 (0.78–9.46)	
**LVEDD** **(female < 51 mm)**	3.02 (2.33–4.15)	0.190	115.5 (90.1–163.8)	0.989	0.92 (0.41–4.52)	0.489
**LVEDD** **(female > 51 mm)**	2.79 (1.78–3.95)		135.6 (78.6–169.8)		1.61 (0.53–7.31)	
**LVESD** **(male < 40 mm)**	3.56 (2.59–6.26)	0.291	133.1 (93.5–152.4)	0.771	1.34 (0.57–6.95)	***0.032***
**LVESD** **(male > 40 mm)**	3.06 (2.20–4.60)		112.2 (90.1–161.1)		2.96 (1.24–12.30)	
**LVESD** **(female < 35 mm)**	3.0 (2.06–3.94)	0.971	127.9 (84.7–162.4)	0.793	0.72 (0.36–3.13)	***0.024***
**LVESD** **(female > 35 mm)**	2.89 (1.93–4.09)		130.1 (92.5–169.9)		2.30 (0.56–8.29)	
**TAPSE < 17 mm**	3.42 (2.52–6.16)	***0.040***	140.4 (99.3–167.0)	0.100	3.41 (0.82–10.0)	***0.023***
**TAPSE > 17 mm**	2.89 (2.0–4.14)		115.5 (82.5–158.7)		1.35 (0.53–4.65)	
**Dyspnea at rest—yes**	3.75 (2.84–5.92)	<***0.001***	141.0 (104.8–169.7)	***0.028***	3.61 (0.99–10.84)	<***0.001***
**Dyspnea at rest—no**	2.66 (1.96–4.04)		112.5 (82.7–152.0)		1.13 (0.42–4.39)	
**Exertional dyspnea—yes**	3.19 (2.27–4.45)	0.070	131.1 (92.0–164.4)	0.100	1.79 (0.56–7.36)	0.304
**Exertional dyspnea—no**	2.32 (1.83–2.83)		98.4 (77.5–110.8)		1.26 (0.56–7.11)	
**Cardiac arrhythmias—yes**	3.31 (2.51–4.53)	0.163	124.0 (93.8–161.8)	0.862	2.87 (0.89–8.70)	***0.016***
**Cardiac arrhythmias—no**	2.84 (1.97–4.20)		127.1 (93.8–161.8)		1.13 (0.52–4.53)	

EF—ejection fraction; LVEDD—left ventricular end-diastolic diameter; LVESD—left ventricular end-systolic diameter; NYHA—New York Heart Association; PASP—pulmonary artery systolic pressure; and TAPSE—tricuspid annular plane systolic excursion.

**Table 3:** Differences in NLR, PLR and CAR values depending on patients’ nutritional status and the results of laboratory testing.

Factor	NLR (IQR)	*p*	PLR (IQR)	*p*	CAR (IQR)	*p*
**ALB < 3.2 g/dL**	3.55 (2.66–6.25)	***0.004***	133.7 (100.7–181.8)	0.094	5.93 (1.30–11.50)	<***0.001***
**ALB > 3.2 g/dL**	2.77 (2.02–4.04)		115.3 (89.7–157.4)		1.06 (0.46–2.83)	
**CRP > 10 mg/dL**	3.67 (2.79–5.37)	<***0.001***	135.6 (97.4–177.0)	***0.046***	8.96 (6.20–15.32)	<***0.001***
**CRP< 10 mg/dL**	2.65 (1.88–3.89)		111.1 (82.1–158/0)		0.80 (0.36–1.28)	
**IL-6 > 7 pg/mL**	4.08 (3.04–6.04)	<***0.001***	135.6 (93.6–174.6)	0.317	8.36 (5.17–20.45)	<***0.001***
**IL-6 < 7 pg/mL**	2.63 (1.81–3.73)		119.3 (90.1–165.6)		0.70 (0.39–2.10)	
**TNF-α > 4.24 pg/mL**	4.16 (2.47–5.92)	***0.009***	147.3 (93.6–195.9)	0.105	7.87 (3.1–14.8)	<***0.001***
**TNF-α < 4.24 pg/mL**	2.71 (1.96–3.48)		110.6 (79.6–156.7)		0.85 (0.41–2.09)	
**NT-proBNP > 2800 pg/mL**	3.59 (2.61–6.0)	<***0.001***	127.1 (95.1–168.6)	0.229	3.58 (1.09–9.37)	<***0.001***
**NT-proBNP < 2800 pg/mL**	2.67 (1.90–3.72)		123.0 (82.5–153.9)		0.95 (0.40–3.24)	
**HGB < 12 g/dL**	3.35 (2.43–4.36)	0.438	146.6 (106.3–173.8)	***0.007***	4.79 (0.85–9.53)	***0.002***
**HGB > 12 g/dL**	2.91 (2.16–4.45)		110.6 (80.7–153.7)		1.24 (0.49–3.96)	
**FM< 25.8 kg**	3.59 (2.56–5.06)	***0.016***	124.8 (95.5–161.3)	0.328	2.65 (0.87–9.06)	0.119
**FM > 25.8 kg**	2.67 (1.94–4.12)		111.9 (74.6–158.2)		1.44 (0.53–5.95)	
**FFM < 53.7 kg**	3.36 (2.37–5.06)	0.127	145.3 (97.0–188.0)	***0.011***	2.07 (0.97–6.18)	0.535
**FFM > 53.7 kg**	2.84 (1.96–4.11)		104.3 (76.7–120.7)		1.33 (0.48–9.03)	
**SGA-A**	2.81 (2.16–4.11)	0.107	128.6 (90.1–165.6)	0.637	0.96 (0.38–2.21)	<***0.001***
**SGA-B and C**	3.62 (2.25–5.64)		124.0 (91.6–161.6)		3.57 (1.07–10.0)	
**NRS-2002 < 3**	2.72 (1.97–4.0)	<***0.001***	111.9 (82.5–157.2)	***0.021***	1.13 (0.52–3.08)	<***0.001***
**NRS-2002 > 3**	4.08 (2.90–5.78)		144.2 (106.3–177.5)		7.56 (1.35–12.66)	

ALB—albumin; CRP—C-reactive protein; FFM—fat-free mass; FM—fat mass; HGB—hemoglobin; NRS—Nutritional Risk Score; and SGA—Subjective Global Assessment.

**Table 4:** Significant predictors of the higher NYHA classification in the studied CHF patients—the result of multiple regression analysis.

Factor	Coefficient	Standard Error	r Partial	*p*
**EF%**	−0.019	0.006	−0.333	<0.001
**Dyspnea at rest**	1.10	0.137	0.529	<0.001
**CAR**	0.017	0.008	0.289	0.029
**NT-proBNP**	0.001	0.001	0.282	0.031

## Data Availability

The data presented in this study are available on request from the corresponding author. The data are not publicly available due to privacy and ethical reasons.

## Supplementary Materials

The following supporting information can be downloaded at: https://www.mdpi.com/article/10.3390/jcm13020433/s1. Figure S1: Overall survival in CHF patients depending on median NLR value (A) and NLR value within Q1 + Q2 and Q3 (B) as well as overall survival in CHF patients depending on median PLR value (C) and PLR value within Q1 + Q2 and Q3 (D). Figure S2: Overall survival in CHF patients depending on combination of: median CAR and NLR values (A), CAR and NLR values within Q1 + Q2 and Q3 (B) as well as overall survival in CHF patients depending on combination of median CAR, NLR and PLR values (C) and CAR, NLR and PLE values within Q1 + Q2 and Q3 (D).
