# Supplementary material for: Correlation between Neutrophil-to-Lymphocyte Ratio, Platelets-to-Lymphocyte Ratio, C-Reactive Protein-to-Albumin Ratio and Clinical Picture of Elderly Chronic Heart Failure Patients
Source: J Clin Med. 2024 Jan 12;13(2):433. doi: 10.3390/jcm13020433 (PMC10817038; doi:10.3390/jcm13020433)
Supplement: Supplementary file 1 [file jcm-13-00433-s001.zip › jcm-2788296-supplementary.pdf]

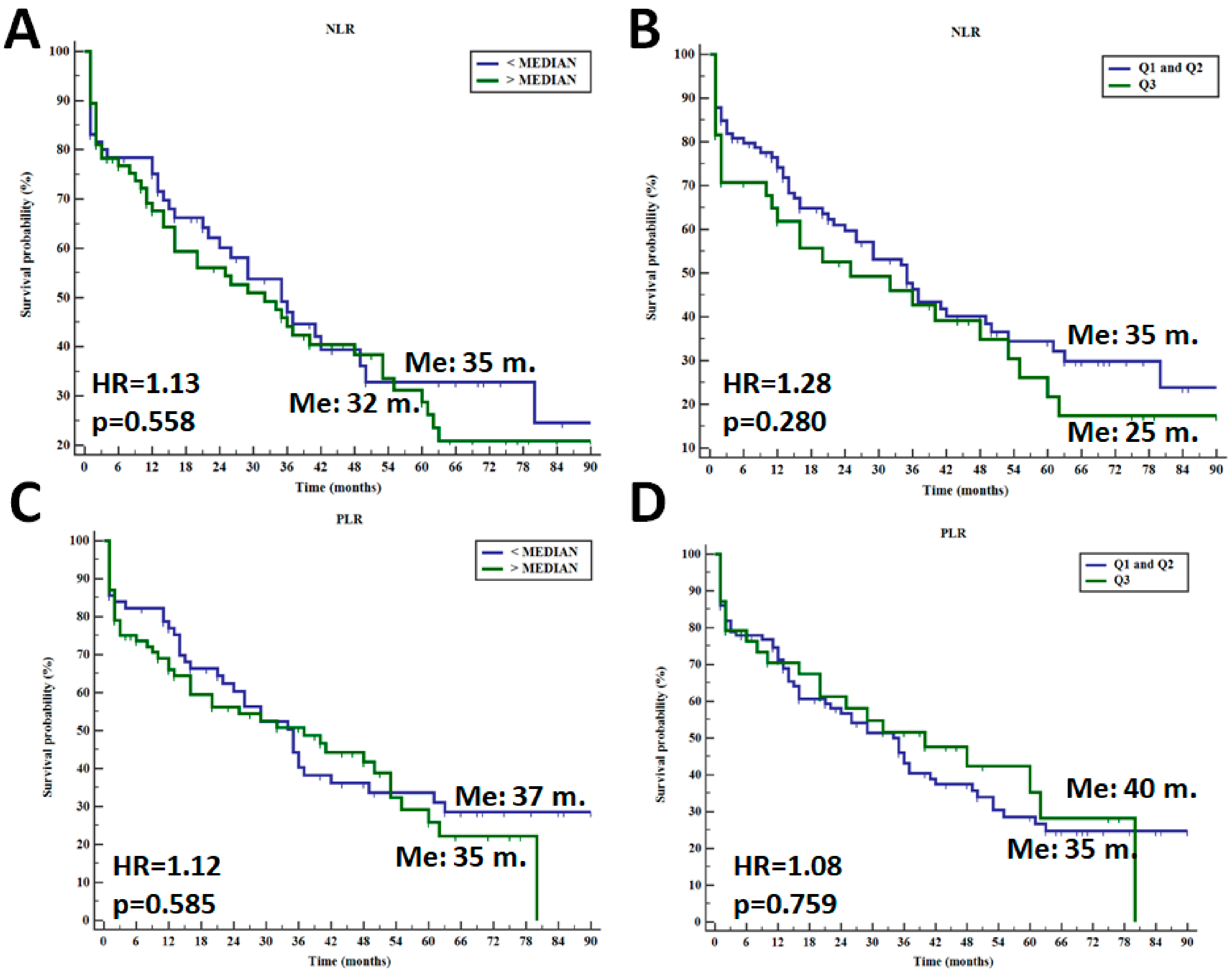

Supplementary Figure S1. Overall survival in CHF patients depending on median NLR value (A) and NLR value within Q1+Q2 and Q3 (B) as well as overall survival in CHF patients depending on median PLR value (C) and PLR value within Q1+Q2 and Q3 (D)

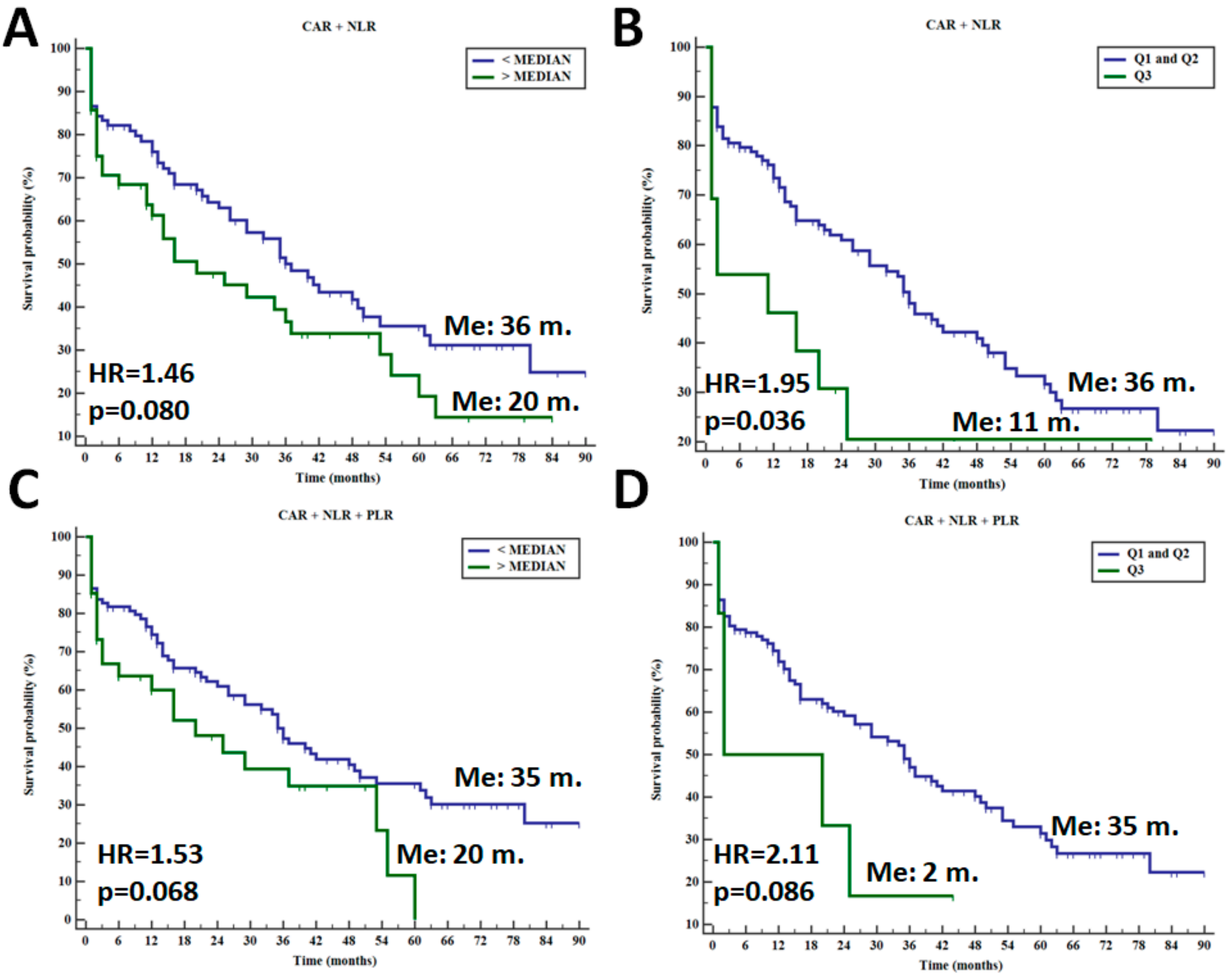

Supplementary Figure S2. Overall survival in CHF patients depending on combination of: median CAR and NLR values (A), CAR and NLR values within Q1+Q2 and Q3 (B) as well as overall survival in CHF patients depending on combination of median CAR, NLR and PLR values (C) and CAR, NLR and PLE values within Q1+Q2 and Q3 (D)
